# Mechanical Properties and Constitutive Model of High-Mass-Fraction Pressed Tungsten Powder/Polytetrafluoroethylene-Based Composites

**DOI:** 10.3390/polym17030323

**Published:** 2025-01-24

**Authors:** Weihang Li, Wenjin Yao, Wei Zhu, Wenbin Li, Bihui Hong, Xinbo Wang

**Affiliations:** School of Mechanical Engineering, Nanjing University of Science and Technology, Nanjing 210094, China; lwh2020@njust.edu.cn (W.L.); 12021059@njust.edu.cn (W.Z.); lwb2000cn@njust.edu.cn (W.L.); hbh2022@njust.edu.cn (B.H.); wangxb@njust.edu.cn (X.W.)

**Keywords:** polytetrafluoroethylene, tungsten powder, compression test, mechanical properties, constitutive model

## Abstract

Heavy metal powders driven by explosions can enhance the near-field lethality of explosive warheads by forming a quasi-pressure field while reducing collateral damage at medium and long ranges. Incorporating polymers into high-content metal powders prevents powder sintering under explosive high pressure, enhancing dispersion uniformity and making them promising for controllable warhead applications. To describe the mechanical behavior of materials under impact loading, this paper investigates the dynamic and static mechanical properties and constitutive modeling of tungsten powder/polytetrafluoroethylene (PTFE) composites. Quasi-static compression tests and split Hopkinson pressure bar (SHPB) dynamic tests were conducted on composites with varying tungsten contents (0 wt%, 70 wt%, 80 wt%, and 90 wt%) and particle sizes (200 μm, 400 μm, and 600 μm), obtaining compressive stress–strain curves over a strain rate range of 0.001 to 3610 s^−1^. The compressive strength of the composites slightly decreased with increasing tungsten particle size but increased with higher tungsten content. Under quasi-static compression, the compressive strength of the composites with 70 wt% and 80 wt% tungsten was lower than that of pure PTFE. This was due to the bonding strength between the tungsten particles and the resin being weaker than the cohesion within the resin. Additionally, the random distribution of the tungsten particles in the matrix led to shear cracks propagating along the phase interfaces, reducing the compressive strength. The compressive strength of the composites with 90 wt% tungsten exceeded that of pure PTFE, as the packed arrangement of the tungsten particles increased the material strength through particle extrusion and friction during compression. Under dynamic impact, the compressive strength of the composites was higher than that of pure PTFE, primarily due to particle extrusion and friction effects. The composites exhibited significant strain rate sensitivity, with both the compressive strength and critical strain increasing quasi-linearly with the strain rate. Based on the experimental data, a damage-modified Zhu–Wang–Tang (ZWT) viscoelastic model was employed to fit the data, effectively characterizing the uniaxial compressive constitutive behavior of tungsten powder/PTFE composites.

## 1. Introduction

Fragmentation bombs generate high-speed metal fragments at the millimeter scale, which exhibit extremely high lethality against enemy personnel within the kill radius [1]. However, outside the effective kill range, these fragments, while unable to completely eliminate living targets, still pose a significant risk of collateral damage to nearby civilians and infrastructure. Replacing millimeter-scale fragments with sub-millimeter heavy metal powder damage elements, such as tungsten or iron powders, can significantly reduce the likelihood of collateral damage [2,3]. In the explosive near-field, metal powders, due to their high-speed and high-density dispersal characteristics, form a quasi-pressure field that enhances the destructive power of ammunition [4]. Meanwhile, the substantial air resistance they encounter causes rapid deceleration, resulting in minimal harm to living targets in the mid- to far-field [5]. These characteristics, particularly their low-collateral-damage (LCD) nature, have led to their widespread adoption in the design of controllable lethality munitions.

The existing research on the characteristics of LCD warheads primarily focuses on the dispersal properties of metal powders under explosive conditions [3,6,7,8]. Chen et al. [6] analyzed the kinetic characteristics, including the number density, velocity, and distribution, of metal powder clouds formed under explosive drive, and found that metal particles are prone to sintering under the high-temperature and high-pressure conditions of detonation, leading to an uneven spatial distribution. Bai et al. [7] proposed adding dispersants to metal powders to address this issue. Li et al. [8] conducted explosive tests on metal powder/polymer-based composites formed by mixing polytetrafluoroethylene (PTFE) with tungsten powder and similarly found that adding PTFE as a dispersant in tungsten powder can effectively prevent sintering, significantly reducing powder agglomeration and improving the uniformity of powder dispersal. Therefore, compared to pure metal powders, metal powder/polymer-based pressed composites show more promising potential for use in LCD warheads. However, research on the dynamic mechanical properties of these composites remains limited, hindering their application in the system design of related warheads.

Metal powder/polymer-based composites are not only suitable for LCD warheads but also have broad applications in energetic reactive fragments, typically fabricated using sintering processes [9]. Many researchers [10,11,12,13,14,15] have investigated the static and dynamic mechanical behaviors and constitutive models of these composites. Wu et al. [10,11] prepared Al/PTFE composites with different Al particle sizes, using quasi-static compression and impact hammer tests to characterize the mechanical properties and reactivity of the materials. They also added Ni to Al/PTFE composites to enhance the energy density and damage effects, analyzing the mechanical performance and response characteristics of the specimens. Feng et al. [12] studied the mechanical properties of Al/PTFE specimens under quasi-static compression and found that excessive Al particles can disrupt the continuity of the PTFE matrix, leading to a decrease in mechanical strength and reactivity. Wang et al. [13] investigated the impact of different tungsten (W) contents on the mechanical behavior of energetic W/PTFE/Al composites. Ding et al. [14] examined the quasi-static and dynamic mechanical properties of PTFE/Al/Si reactive materials and developed a Johnson–Cook constitutive model considering the strain hardening, strain rate hardening, and thermal softening effects. Sun et al. [15] studied the mechanical properties, constitutive behavior, and failure criteria of Al/PTFE/W reactive materials and determined the Johnson–Cook constitutive model and failure parameters through experimental data and numerical iteration. Tang et al. [16] used a cold-press rapid cooling process to prepare reinforced Al/PTFE reactive materials and tested their dynamic mechanical properties at various strain rates using the SHPB test system, calibrating the Zhu–Wang–Tang (ZWT) constitutive model parameters. These studies provide valuable insights into the dynamic mechanical behavior of composites used in LCD warheads.

However, it is important to note that the composite materials used in LCD warheads and energetic fragments differ not only in their preparation processes but also significantly in the size and mass ratio of the metal powders. To achieve an effective shock-induced thermal energy release, the metal powders in energetic fragments are typically nanoscale or microscale, with a relatively low mass fraction (e.g., Al powder and PTFE powder are commonly mixed at a mass ratio of 23.5:76.5 [17]). In contrast, the composite materials used for the shells of LCD warheads typically consist of metal powders in the millimeter scale, with the mass fraction of metal powders often exceeding 50% [7,8], aiming to enhance the near-field pressure field through a dense powder cluster. These differences suggest that the dynamic mechanical properties of the metal powder/polymer composites used in LCD warheads are likely to differ substantially from those of energetic metal-based composites.

Therefore, this study focuses on tungsten powder/poly(tetrafluoroethylene) (PTFE) composites with a high metal content and employs both a universal testing machine and the SHPB device to perform quasi-static and dynamic compression tests to obtain their mechanical properties. The deformation behavior and strain rate effects of tungsten powder/PTFE composites with tungsten contents of 0 wt%, 70 wt%, 80 wt%, and 90 wt% and tungsten particle sizes of 200 μm, 400 μm, and 600 μm were investigated over strain rates ranging from 0.001 to 3610 s^−1^. A damage-correction-based ZWT viscoelastic constitutive model was developed to accurately describe the mechanical response of the composite material under dynamic loading conditions.

## 2. Experimental Procedures

### 2.1. Materials and Sample Preparation

**Figure 1:**
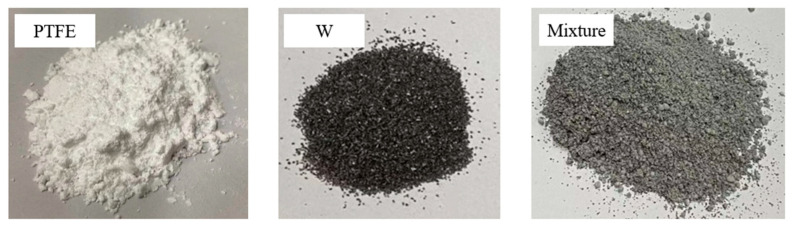
The morphology of polytetrafluoroethylene powder, tungsten powder, and a mixture of the two powders.

The raw materials for specimen preparation are shown in Figure 1. Specimens with varying compositions and particle sizes were prepared using 40 μm polytetrafluoroethylene (PTFE) resin powder and tungsten powder with particle sizes of 200 μm, 400 μm, and 600 μm as reinforcing fillers. PTFE was manufactured by Tianyuxiang Micro Powder Material Factory in Shenyang, China. Tungsten powder was manufactured by Mudanjiang North Alloy Tools Co., Ltd. in Mudanjiang, China. Material preparation primarily consisted of two stages: the PTFE-tungsten powder mixing stage and the compaction stage. First, the PTFE resin powder and tungsten powder were placed in a powder mixer and uniformly blended for 15 min using the mixer’s rotation. The blended powder was then quantitatively placed into uniaxial die compaction equipment. A press was used to compact the powder at a rate of 0.1 mm/s until the powder bonded into shape and achieved the desired density. After reaching the target pressure, the pressure was maintained for 1 min. After compaction, the specimens were left in a constant-temperature chamber (25 °C) for 24 h to eliminate residual stress. Both quasi-static and dynamic test specimens were prepared using this method.

**Figure 2:**
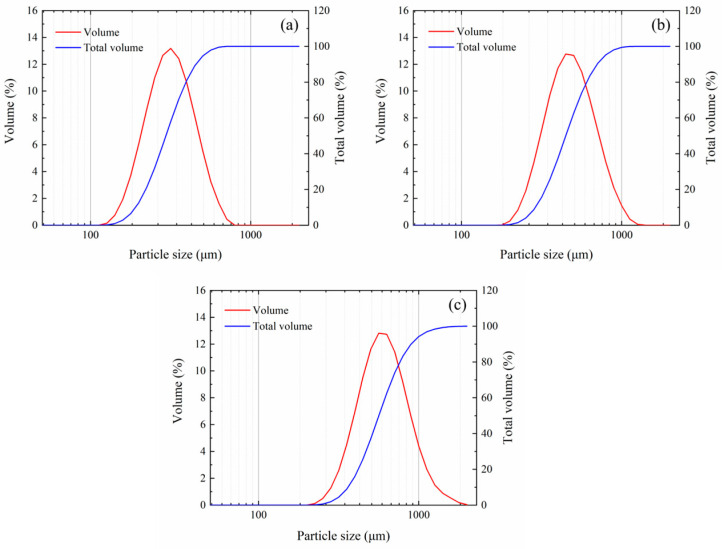
Particle size frequency distribution and cumulative distribution of tungsten powder with particle sizes of (**a**) 200 μm, (**b**) 400 μm, and (**c**) 600 μm.

The particle size distributions of the three tungsten powder types were measured using a Mastersizer 2000 laser particle size analyzer from Malvern Panalytical, Malvern, UK. The results shown in Figure 2. The results indicate that the tungsten powder had a relatively narrow particle size distribution. Table 1 presents the measured typical particle size values for the different tungsten powder specifications. For example, for the 400 μm tungsten powder, Figure 2 shows that over 80% of the tungsten powder particles had sizes ranging from 350 to 460 μm. The median particle sizes (D50) of the three tungsten powder specifications were found to be 237 μm, 422 μm, and 609 μm, respectively.

**Figure 3:**
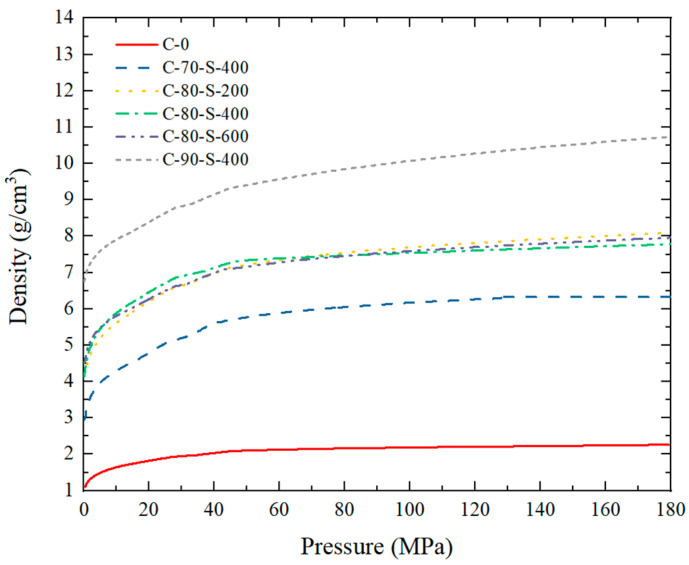
Bulk density of mixed tungsten powder and PTFE powder with different contents and particle sizes under various pressures.

The specimen identification codes and corresponding schemes are shown in Table 2. The specimens with weight ratios of 7:3, 8:2, and 9:1 of 400 μm tungsten powder to resin powder were labeled as C-70-S-400, C-80-S-400, and C-90-S-400, respectively. The specimens with a weight ratio of 8:2 of tungsten powder to resin powder and tungsten powder particle sizes of 200 μm, 400 μm, and 600 μm were labeled as C-80-S-200, C-80-S-400, and C-80-S-600, respectively. The specimen made of pure resin powder was labeled as C-0. The theoretical density of the specimens was determined by the weight ratio of tungsten powder to resin powder, with theoretical densities of 5.69 g/cm^3^, 7.44 g/cm^3^, and 10.75 g/cm^3^ for C-70, C-80, and C-90, respectively. The density of the compacted powder was influenced by the applied pressure. As the pressure exceeded a certain threshold, its effect on the density became less significant with further increases in pressure, as shown in Figure 3.

**Figure 4:**
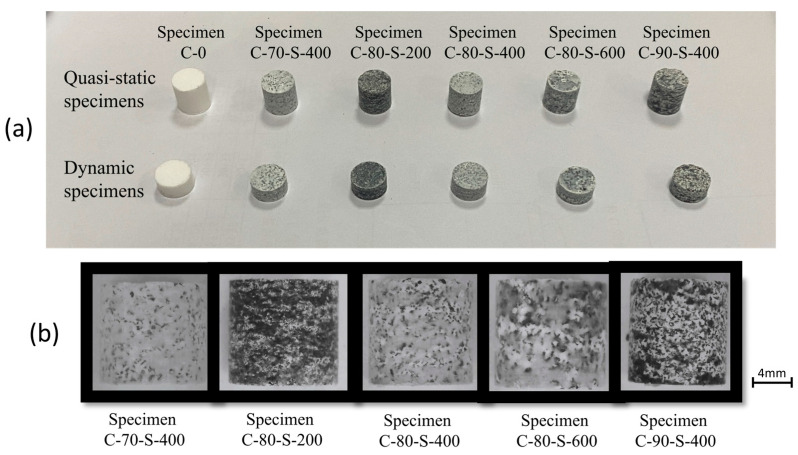
Actual images of the samples: (**a**) quasi-static and dynamic loading specimens; (**b**) microstructure of the samples.

For the quasi-static loading, the specimen had both a diameter and thickness of 10 mm. In the dynamic testing, reducing the specimen thickness appropriately enhanced the signal transmission and ensured stress equilibrium. However, an excessively small length-to-diameter ratio increased the frictional effects at both ends of the specimen; thus, dynamic loading specimens with a diameter of 10 mm and a thickness of 5 mm were used. The quasi-static and dynamic loading specimens are shown in Figure 4. The images reveal that the particles were evenly distributed, with no significant agglomeration or clustering observed. This confirms that the blending process was effective in achieving uniform dispersion.

### 2.2. Quasi-Static Testing

The quasi-static testing was performed on the WGD-1 MTS universal testing machine made in Song Ying Instrument Manufacturing Co., Ltd., Shanghai, China. The compression speed of the testing machine was set to 0.6 mm/min, corresponding to a strain rate of 1 × 10^−3^ s^−1^. The engineering stress and strain of the specimens were calculated based on the compression load data from the sensors and displacement data from the crosshead, and the stress–strain curves were constructed. Each test was repeated three times, and the average value of the results was taken.

### 2.3. Dynamic Testing and Improvement of SHPB Method

The split Hopkinson pressure bar (SHPB) apparatus is commonly used to study the mechanical behavior of materials under high strain rates, and many studies on the mechanical behavior of polymer-based materials have utilized this device [18,19,20]. Advances in pulse-shaping techniques [21,22] have enabled waveform control, enabling stress equilibrium and facilitating constant strain loading through the selection of appropriate pulse shaper materials and dimensions [23,24].

**Figure 5:**
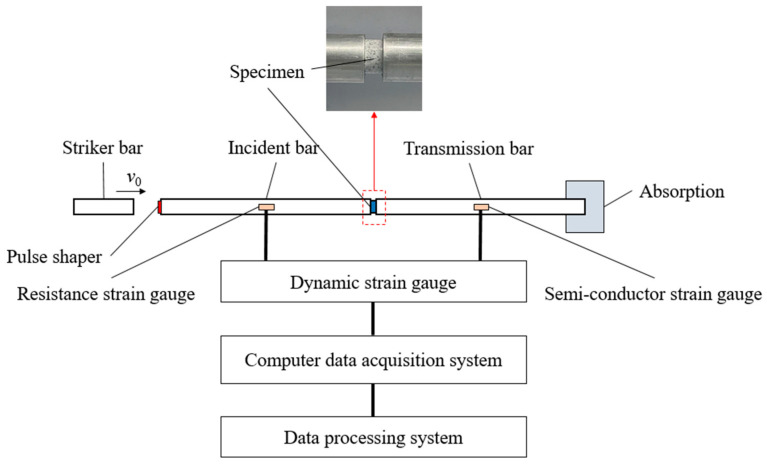
Schematic diagram of the SHPB apparatus.

Dynamic testing was conducted using a split Hopkinson pressure bar (SHPB) system. A schematic of the system is shown in Figure 5. The SHPB system consisted of a 14.5 mm diameter aluminum projectile, an incident bar, a transmission bar, and an energy absorber. The projectile used had a length of 200 mm, and both the incident and transmission bars had lengths of 1300 mm. Semiconductor strain gauges attached to the incident and transmission bars recorded the strain signals, with the strain gauges positioned at the midpoints of the bars. The Wheatstone bridge, constructed from the strain gauges, converted the strain signals from the bars into voltage signals. These signals were then amplified by high-dynamic resistance strain gauges and processed by a data acquisition card. The voltage–time curves for the two strain gauges were displayed on the monitor connected to the data acquisition card.

**Figure 6:**
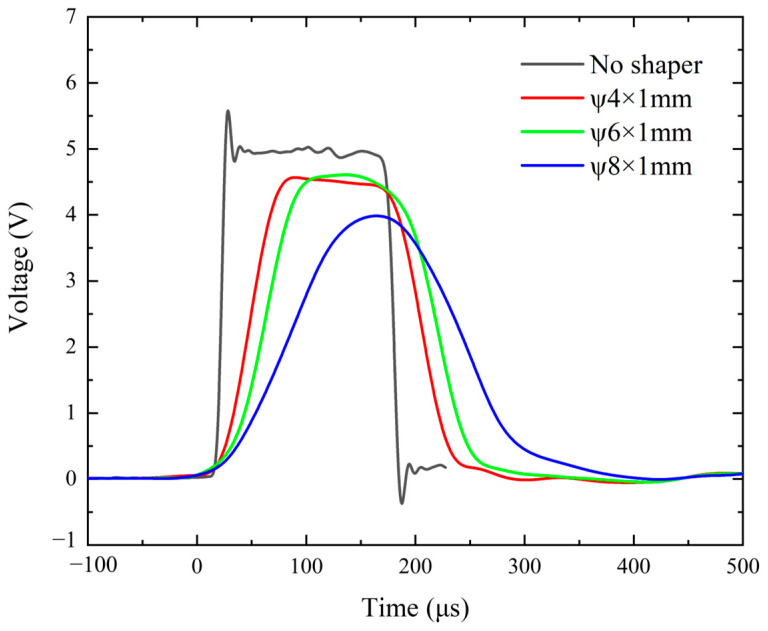
Incident signals under different pulse-shaping conditions. The black line represents the incident waveform without shaping, while the red, green, and blue lines correspond to waveforms shaped using silicone rubber shapers with diameters of 4 mm, 6 mm, and 8 mm, and a thickness of 1 mm, respectively.

**Figure 7:**
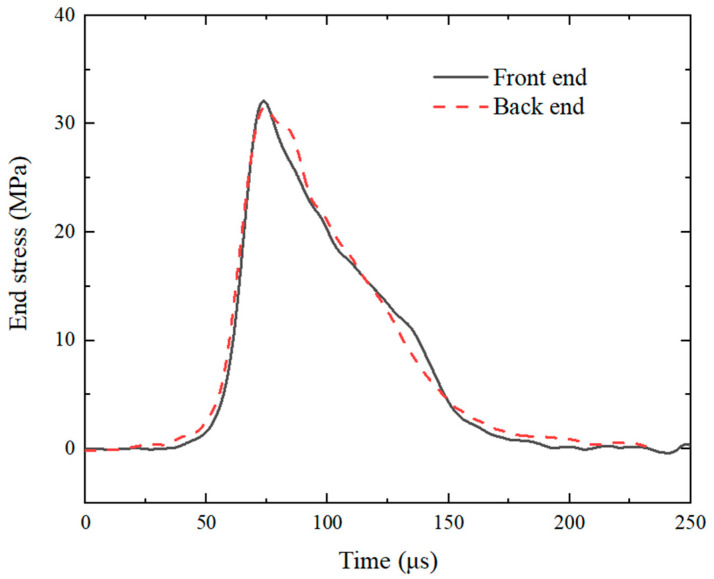
Stress vs. time curves at two ends of specimen.

To ensure stress equilibrium and uniform deformation of the specimen during testing, cylindrical silicone rubber pulse shapers of varying sizes were affixed between the projectile and the incident bar. A comparison of the waveforms generated using pulse shapers of different sizes is shown in Figure 6. With the addition of the pulse shaper, the rise time of the incident waveform increased from 17.99 μs to 75.46 μs, 93.46 μs, and 148.78 μs. Additionally, the silicone rubber pulse shaper significantly reduced the oscillations of the incident waveform. The inclusion of the pulse shaper noticeably improved the incident waveform. As shown in Figure 7, the stress vs. time curves at the two ends of specimen are in good agreement; specifically, the assumption of dynamic stress equilibrium was experimentally verified.

To enhance signal transmission, LC4 ultra-high-strength aluminum alloy bars with a density of 2700 kg/m^3^ and an elastic modulus of 71 GPa were used to reduce the impedance mismatch between the specimen and the transmission bar. Additionally, semiconductor strain gauges were incorporated to improve the signal-to-noise ratio of the transmitted wave signal. Molybdenum disulfide was also applied as a lubricant to both ends of the specimen to reduce the end-face friction caused by the small length-to-diameter ratio of the specimen. Impact tests were conducted at strain rates ranging from 920 to 3610 s⁻^1^, with each test repeated three times and the average value taken. Based on the one-dimensional and uniformity assumptions, Equations (1)–(3) were used to determine the engineering stress, engineering strain, and strain rate [25]:

(1) $$\sigma (t)=\frac{S_{0}}{S_{S}}E\varepsilon_{t}(t)$$

(2) $$\varepsilon (t)=-\frac{2c_{0}}{L_{S}}\int_{0}^{t}\varepsilon_{r}(t)d\tau$$

(3) $$\dot{\varepsilon }(t)=-\frac{2c_{0}}{L_{S}}\varepsilon_{r}(t)$$

Here, *ε_t_*(*t*) and *ε_r_*(*t*) represent the transmitted strain and reflected strain, respectively; *E*, *S*_0_, and *c*_0_ represent the Young’s modulus, cross-sectional area, and longitudinal wave velocity in the incident and transmission bars, respectively; and *S_S_* and *L_S_* denote the initial cross-sectional area and length of the specimen, respectively. Furthermore, the true stress–strain relationship of the specimen can be calculated using Equations (4)–(6) [25].

(4) $$\sigma {\left(t\right)}_{t}=(1-\varepsilon (t))\cdot \sigma (t)$$

(5) $$\varepsilon {\left(t\right)}_{t}=-\ln(1-\varepsilon (t))$$

(6) $$\dot{\varepsilon }{\left(t\right)}_{t}=\frac{\dot{\varepsilon }(t)}{1+\varepsilon (t)}$$

In the equations, $\sigma {\left(t\right)}_{t}$, $\varepsilon {\left(t\right)}_{t}$, and $\dot{\varepsilon }{\left(t\right)}_{t}$ represent the true stress, true strain, and true strain rate, respectively.

## 3. Results and Discussion

### 3.1. Quasi-Static Testing

**Figure 8:**
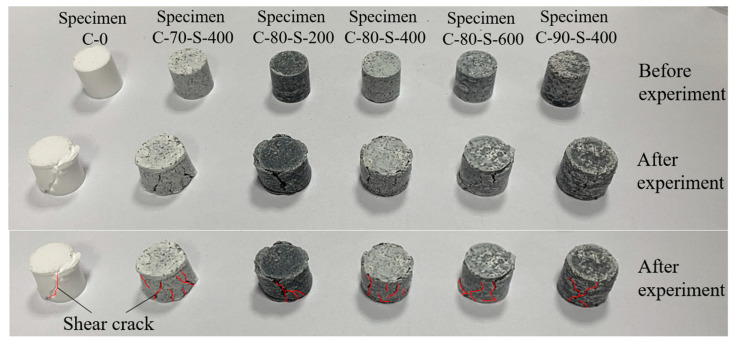
Photographs of specimens after quasi-static compression tests, with red lines indicating the disordered crack propagation paths.

Figure 8 illustrates the deformation of the specimens after the quasi-static tests, showing that cracks formed on the lateral surfaces after compression. For the C-0 specimen, cracks extended at a 45° angle relative to the axial direction after compression, whereas the specimens containing tungsten powder exhibited disordered crack propagation. The number of cracks decreased with increasing tungsten content but increased with larger tungsten particle sizes. This demonstrates that with high tungsten content, the response of the tungsten powder/PTFE-based composites under low strain rates differed significantly from the resin matrix, with tungsten powder dominating the material’s mechanical behavior.

**Figure 9:**
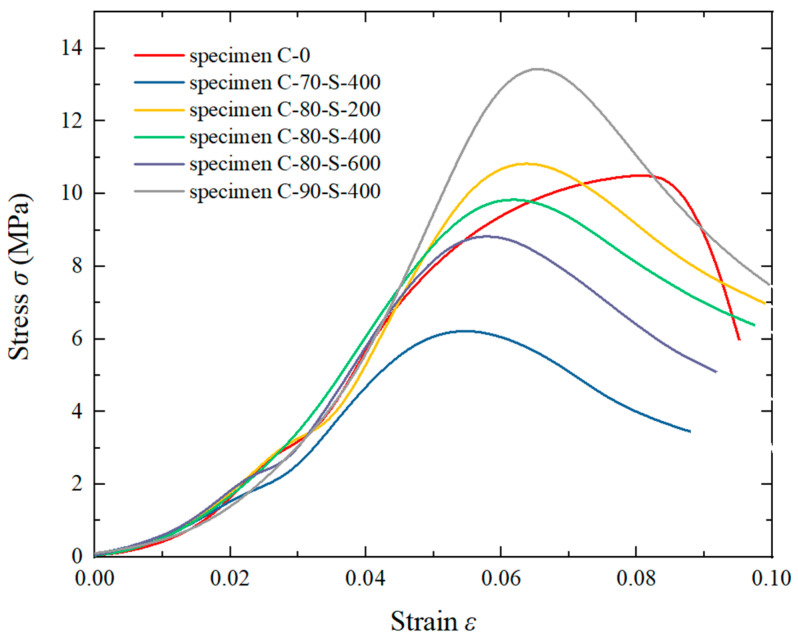
Stress–strain curve of the quasi-static test.

Figure 9 shows the stress–strain curves of the composites under quasi-static testing. Initially, the material underwent linear elastic deformation, followed by nonlinear deformation, leading to a stress peak. Then, it transitioned into a strain-softening phase, and finally reached an expansion–fracture stage. The critical strain is defined as the strain value corresponding to the ultimate strength of the material. The stress–strain curves reveal that the mechanical response of C-0 differed from that of the tungsten powder-containing composites under quasi-static conditions. The addition of tungsten powder reduced the critical strain of the resin, and the stress decreased more rapidly after specimen failure. This indicates that the pure resin rapidly lost its strength upon cracking but retained some compressive capacity after reaching the stress peak.

Additionally, it was observed that the ultimate strengths of C-70 and C-80 were lower than that of pure resin. At a low tungsten powder content, the bonding strength between the tungsten particles and the resin was weaker than the inter-resin bonding. The disordered distribution of the tungsten powder in the matrix led to the propagation of shear cracks along the two-phase interface during compression. When the tungsten powder content exceeded a certain threshold, the packing and arrangement of the tungsten particles enhanced the overall material strength through particle compaction and friction during compression, causing the ultimate strength of C-90 to exceed that of pure resin.

With a particle size of 400 μm, the ultimate strengths of C-70, C-80, and C-90 were 6.21 MPa, 9.84 MPa, and 13.44 MPa, respectively. Increasing the tungsten powder content enhanced the particle-to-particle contact, indirectly improving the load-bearing capacity of the tungsten powder. At 80% tungsten content, the ultimate strengths of S-200, S-400, and S-600 were 10.83 MPa, 9.84 MPa, and 8.82 MPa, respectively, with the ultimate strength decreasing as the particle size increased. Larger tungsten particles facilitated the propagation of micro cracks within the matrix, whereas smaller particles were more effective in impeding crack growth.

### 3.2. Dynamic Testing

**Figure 10:**
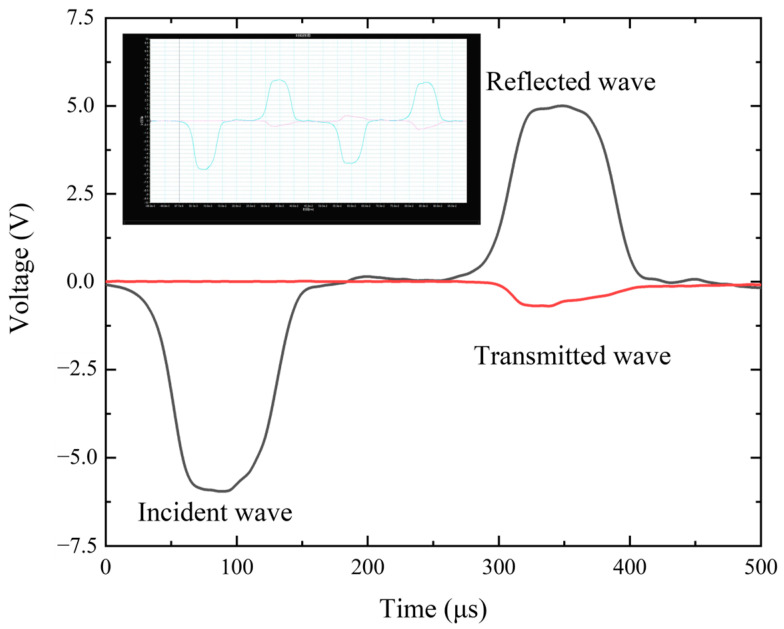
Raw data from the SHPB test.

Figure 10 shows a set of typical SHPB test waveforms. The green line represents the incident and reflected signals recorded in the incident bar, while the red line represents the transmitted signal recorded in the transmission bar. From the reflected waveform, it can be seen that constant strain rate loading was achieved during the experiment. Additionally, the rise of the transmitted wave slowed down, and the transmitted signal became more distinct, providing a good foundation for determining the stress–strain relationship.

**Figure 11:**
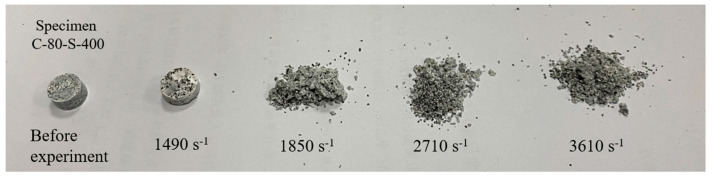
Morphology and fracture behavior of sample C-80-S-400 under different loading rates.

Figure 11 shows the morphology of C-80-S-400 under different loading rates. As the strain rate increased, the specimen’s damage gradually intensified until complete fracture occurred. At low strain rates, the damage range was broader, the cracks were smaller, and the damage degree was lower. The cracks were relatively concentrated in the center region, with random crack initiation and an unordered propagation direction. As the strain rate increased, the specimen underwent complete fragmentation along the crack propagation direction.

**Figure 12:**
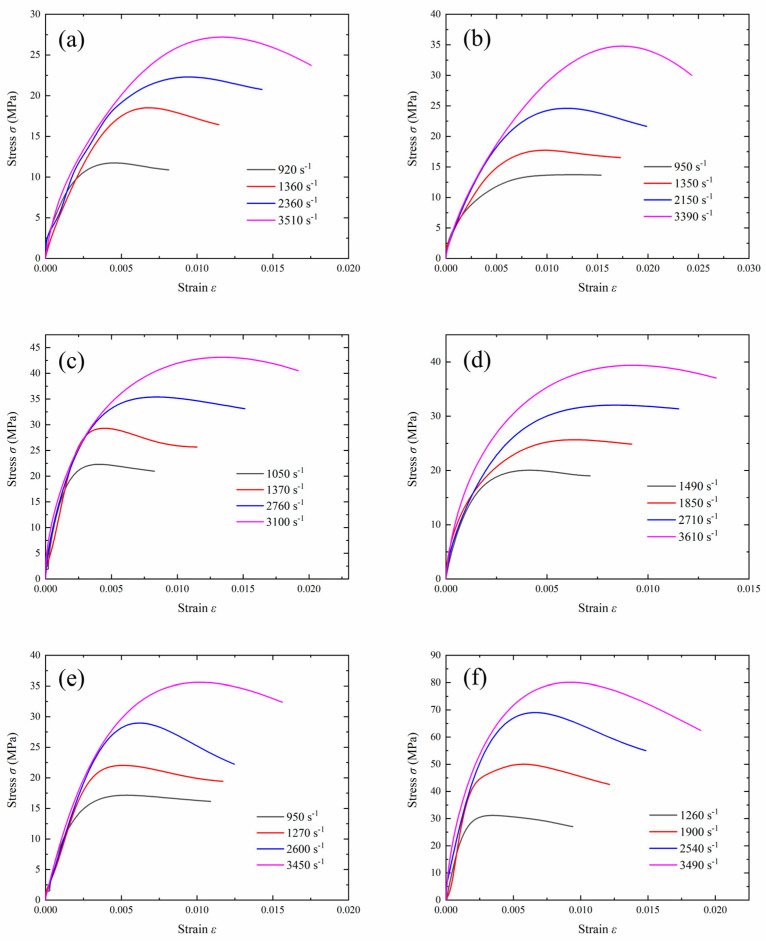
Stress–strain curves under dynamic testing for specimens (**a**) C-0, (**b**) C-70-S-400, (**c**) C-80-S-200, (**d**) C-80-S-400, (**e**) C-80-S-600, and (**f**) C-90-S-400.

Based on the one-dimensional stress wave propagation theory, the double wave method was used to process the experimental data, obtaining the stress–strain curves of the specimen under different strain rates. Figure 12 shows the stress–strain curves of the composite material during dynamic testing. The experimental stress–strain curves do not show a distinct yield plateau. Therefore, the peak stress and corresponding strain values under different strain rates can be obtained from the curves. As the strain rate increased, the peak stress gradually increased.

### 3.3. Effects of Strain Rate, Tungsten Powder Size, and Content on Dynamic Mechanical Properties

**Figure 13:**
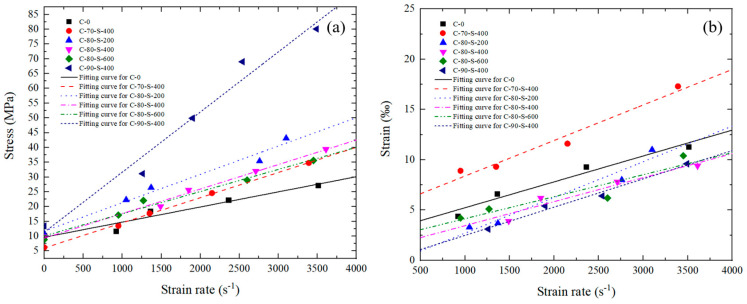
Relationships between strain rate and (**a**) ultimate strength and (**b**) critical strain.

To further evaluate the material’s performance under different loading rates, the ultimate strength and critical strain of the material were characterized. Figure 13 shows the variation curves of the ultimate strength and critical strain with the strain rate. The trend indicates that both the ultimate strength and critical strain increased linearly with the strain rate. As shown in Figure 13a, the ultimate strength was rate-dependent, which implies that the composite material exhibited viscoelastic behavior at low strains.

Compared to the quasi-static experimental data, at high strain rates of 920–3610 s^−1^, the stress in the first elastic phase increased sharply, indicating an interaction between the particles and the matrix under high loads. Under dynamic loading, the strain rate effect was significant for all four specimens, and the degree of hardening increased notably. For example, at a strain rate of 1490 s^−1^, the stress of specimen C-80-S-400 was 20.028 MPa, and at 3610 s^−1^, the stress of specimen C-80-S-400 increased to 39.379 MPa, a 96.6% increase. Both of these stresses were greater than the quasi-static stress of 7.398 MPa. At high strain rates, the molecular chains of the specimen and tungsten particles interacted significantly in a very short time, directly resisting deformation and causing the stress to increase. Additionally, the stress of the C-0 specimen increased linearly in the later stages of deformation, unlike the behavior of the C-70, C-80, and C-90 specimens. Under high strain rate loading, a large number of tungsten particles directly bore the external compressive stress; therefore, the mechanical properties of the specimens were controlled by the properties of the tungsten phase.

Apart from the strain rate, the tungsten powder content and particle size are important parameters influencing a material’s performance under different loads [26]. As the tungsten powder content increased, the ultimate strength and critical strain of the material gradually increased, and the effect of the loading rate on the ultimate strength and critical strain became more pronounced. The tungsten powder content significantly affected the ultimate strength. Tungsten is a highly dense and strong material, so increasing its content generally improved the composite’s ability to withstand compressive forces. More tungsten particles created a stronger reinforcement network within the PTFE matrix, leading to an improvement in the compressive strength. Increasing the tungsten powder content increased the chances of direct contact between particles. The increase in the elastic modulus arose from the inherent hardness provided by the increased number of hard tungsten particles. Additionally, at high strain rates, tungsten particles bore most of the external load, significantly enhancing the ultimate strength and critical strain.

The particle size of the tungsten powder is another critical factor affecting ultimate strength and critical strain. Reducing the tungsten powder particle size slightly increases the ultimate strength, while at high strain rates, the critical strain decreases as the tungsten powder particle size increases. Smaller particle sizes make crack propagation under compression more difficult. Furthermore, smaller particles have a larger surface area and a greater bonding area with the matrix, which enhances the overall strength of the matrix. As the particle size increases, the surface area-to-volume ratio of the individual tungsten particles decreases, which can lead to a weaker particle–matrix interface. The bonding strength between the PTFE matrix and larger tungsten particles tends to be weaker compared to smaller particles, as there is less surface area for effective interaction. Larger particles can also create larger voids or poorly consolidated regions within the matrix, where stress concentrations can lead to premature failure under compression. These factors reduce the overall compressive strength of the composite. Tungsten particles act as fillers within the PTFE matrix, transferring stress during loading. However, larger particles do not integrate as well into the matrix, potentially reducing the efficiency of load transfer. When the particle size increases, the matrix becomes less effective at transferring stress to the tungsten particles, leading to a reduction in the overall material strength.

## 4. Constitutive Model of Tungsten Powder/PTFE Matrix Composites

To facilitate the engineering application of the material in numerical simulations, the Zhu–Wang–Tang viscoelastic constitutive model was adopted, based on the summarized experimental results, to describe the mechanical behavior of W/PTFE composites under uniaxial stress conditions.

### 4.1. ZWT Viscoelastic Constitutive Model with Damage Correction

The composite material consisted of PTFE and tungsten metal particles, with the particles tightly encapsulated by the resin. During impact compression, the W particles underwent sliding, and the resin was compressed, resulting in significant strain. In this process, the resin acted as a matrix or adhesive, leading to the classification of this material as viscoelastic. Various researchers have proposed different constitutive models from diverse perspectives to describe the mechanical properties of polymers under dynamic loading. Examples include the modified Ramberg–Osgood model [27], the geomechanical model [28], and the Visco-SCRAM model [29]. Considering the evident internal defects in W/PTFE under dynamic loading conditions, the ZWT viscoelastic constitutive model [30] is a suitable choice.

**Figure 14:**
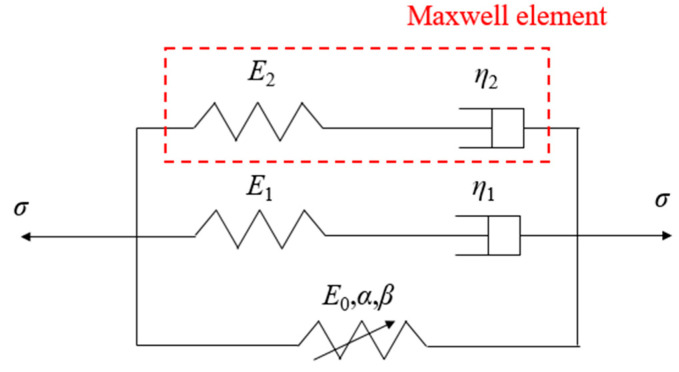
ZWT nonlinear viscoelastic constitutive model.

The ZWT constitutive model is a viscoelastic constitutive model composed of a nonlinear spring in parallel with two Maxwell models (one high-frequency and one low-frequency), as shown in Figure 14. It is suitable for describing the mechanical behavior of materials under both high and low strain rates.

Two Maxwell elements are used to describe the viscoelastic responses under low and high strain rates, respectively, while a nonlinear spring is employed to represent the nonlinear elastic response. The expression of the Zhu–Wang–Tang constitutive model is as follows:

(7) $$\sigma =E_{0}\varepsilon +\alpha \varepsilon^{2}+\beta \varepsilon^{3}+E_{1}\int_{0}^{t}\dot{\varepsilon }(\tau )\exp(-\frac{t-\tau }{\theta_{1}})d\tau +E_{2}\int_{0}^{t}\dot{\varepsilon }(\tau )\exp(-\frac{t-\tau }{\theta_{2}})d\tau$$

Here, *σ* represents the Cauchy stress, *ε* denotes the Cauchy strain, and *E*_0_, *α*, and *β* are the elastic constants of the nonlinear component. *E*_1_, *θ*_1_, *E*_2_, and *θ*_2_ correspond to the elastic constants and relaxation times of the viscoelastic components under low and high strain rates, respectively.

At high strain rates, the duration of the impact experiment is relatively short, and the low-frequency term does not have sufficient time to relax. Therefore, the first Maxwell element can be regarded as a spring, and the elastic modulus *E*_1_ can be combined with *E*_0_. Equation (7) can then be simplified as:

(8) $$\sigma =E_{0}\varepsilon +\alpha \varepsilon^{2}+\beta \varepsilon^{3}+E_{2}\int_{0}^{t}\dot{\varepsilon }(\tau )\exp(-\frac{t-\tau }{\theta_{2}})d\tau$$

In polymer materials, when the strain reaches a certain value, micro cracks visible to the naked eye begin to appear, accompanied by strain softening (*dσ*/*dε*). Experimental observations indicate that the number of micro cracks increases with both the deformation and strain rate. The formation of micro cracks inevitably weakens the material’s resistance to deformation, leading to some form of material weakening or softening. Let *σ_r_* represent the stress of the undamaged material and *σ_a_* represent the stress of the damaged material. The damage evolution model considers the nonlinear relationship between the damage factor and strain, where *D* is the damage factor, expressed as follows:

(9) $$\sigma =[1-D][E_{0}\varepsilon +\alpha \varepsilon^{2}+\beta \varepsilon^{3}+E_{1}\int_{0}^{t}\dot{\varepsilon }(\tau )\exp(-\frac{t-\tau }{\theta_{1}})d\tau +E_{2}\int_{0}^{t}\dot{\varepsilon }(\tau )\exp(-\frac{t-\tau }{\theta_{2}})d\tau ]$$

The expression for *D* is as follows:

(10) $$D=K_{D}\varepsilon^{\left(a-1\right)}{\left(\varepsilon -\varepsilon_{th}\right)}^{b},\varepsilon >\varepsilon_{th}$$

Here, *K_D_* and a are material parameters, and *ε_th_* is the strain threshold. Based on Equations (9) and (10), the damage constitutive model under constant strain rate loading is derived as follows:

(11) $$\sigma =[1-K_{D}{\dot{\varepsilon }}^{\left(a-1\right)}(\varepsilon -\varepsilon_{th})][E_{0}\varepsilon +\alpha \varepsilon^{2}+\beta \varepsilon^{3}+E_{2}\int_{0}^{t}\dot{\varepsilon }(\tau )\exp(-\frac{t-\tau }{\theta_{2}})d\tau ]$$

### 4.2. Data Fitting and Model Verification

**Figure 15:**
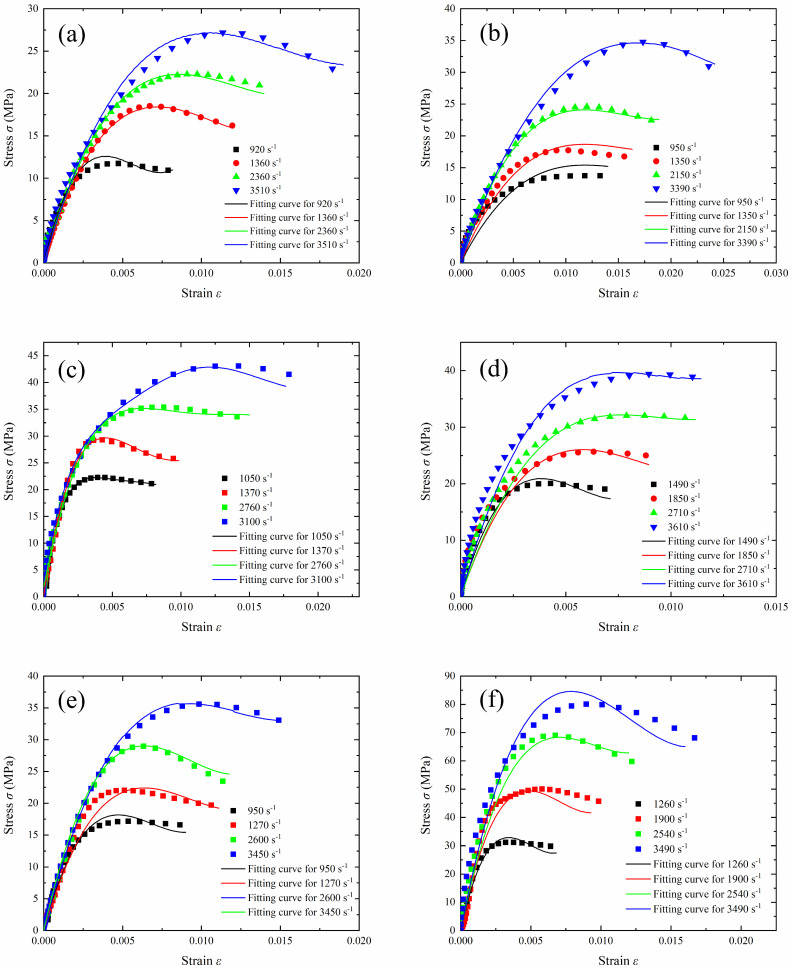
Comparison of the uniaxial stress results for samples (**a**) C-0, (**b**) C-70-S-400, (**c**) C-80-S-200, (**d**) C-80-S-400, (**e**) C-80-S-600, and (**f**) C-90-S-400 at strain rates of 920–3610 s^−1^, with the model predictions. The symbols represent the experimental data, and the lines represent the predictions.

The stress–strain curves at strain rates of 920–3610 s^−1^ are compared in Figure 15; the symbols represent the experimental data, while the lines indicate the predictions of the constitutive model. The fitting results align well with the experimental values. The ZWT viscoelastic constitutive model, modified with damage correction, successfully captures the mechanical response characteristics of the material, including the variations in stress and work hardening with the strain rate. The model constant and R square (R^2^) are listed in Table 2. The R square (R^2^) is used to describe the goodness of fit of the model to the data. For the C-0 sample, the R^2^ value is 0.99, while for the samples containing tungsten powder, the R^2^ values range from 0.92 to 0.97. This demonstrates a strong agreement between the model and the experimental data.

## 5. Conclusions

This study conducted quasi-static and dynamic tests on tungsten powder/PTFE-based composites with different tungsten contents and particle sizes and established a constitutive model to describe their mechanical behavior. The following conclusions are drawn:

The compressive strength of the composite slightly decreased as the tungsten powder particle size increased, while it increased as the tungsten content increased. Under quasi-static compression, the compressive strength of the composites with 70 wt% and 80 wt% tungsten powder was lower than that of pure PTFE. The bonding strength between the tungsten particles and the resin was lower than the bonding strength between the resin molecules, and the disordered filling of the tungsten powder in the matrix led to shear cracks extending along the interface between the two phases when subjected to compression. The compressive strength of the composite with 90 wt% tungsten powder was higher than that of pure PTFE, and the packing arrangement of the tungsten powder inside the composite enhanced the overall strength of the material by squeezing and friction during compression.

The dynamic mechanical properties of this composite were largely dependent on the strain rate, exhibiting significant strain rate sensitivity. Both the compressive strength and the corresponding critical strain increased approximately linearly with the increase in the strain rate. Under quasi-static loading, the 0 wt% sample and the 50 wt%, 70 wt%, and 90 wt% samples exhibited different hardening behaviors. The internal packing arrangement of tungsten powder caused particle compression during the compression process, which significantly enhanced the intermediate hardening stage. At high strain rates, the 90 wt% sample showed significant linear hardening in the late deformation stage, which was due to the mechanical behavior after compression being dominated by the tungsten phase.

A damage-corrected ZWT constitutive model was established, and the corresponding parameters were fitted. The experimental results verify the accuracy of the model, which accurately describes the effect of these parameters on the mechanical behavior of the composite under different strain rates.

## Figures and Tables

**Table 1:** Specimens and experimental schemes.

Serial No.	Specimens	W Mass Fraction (%)	PTFE Mass Fraction (%)	W Particle Size (μm)	*ρ* (g/cm^3^)	Compaction Pressure (MPa)
1	C-0	0	100.00	/	2.15	53.3
2	C-70-S-400	70.00	30.00	400	5.69	45.5
3	C-80-S-200	80.00	20.00	200	7.44	74.9
4	C-80-S-400	80.00	20.00	400	7.44	69.1
5	C-80-S-600	80.00	20.00	600	7.44	74.3
6	C-90-S-400	90.00	10.00	400	10.75	103.9

**Table 2:** Fitted parameter values for the damage-corrected ZWT viscoelastic model.

Specimens	*E*_0_ (MPa)	*α*	*β*	*E*_2_ (MPa)	*θ*_2_	*a*	R Square
C-0	11.096	−2057.521	5.687 × 10^3^	11.708	5.809 × 10^7^	1.440	0.99
C-70-S-400	10.358	−1409.109	3.027 × 10^3^	10.307	6.9404 × 10^7^	1.551	0.97
C-80-S-200	11.218	−2124.696	6.040 × 10^3^	11.222	−1.372 × 10^7^	1.254	0.93
C-80-S-400	10.798	−2510.224	8.610 × 10^3^	10.768	1.671 × 10^7^	1.632	0.98
C-80-S-600	11.968	−2860.741	10.313 × 10^3^	11.954	−2.085 × 10^7^	1.362	0.97
C-90-S-400	5.822	−1328.278	4.316 × 10^3^	5.825	−8.337 × 10^7^	1.811	0.92

## Data Availability

The experimental and numerical modeling results are available upon request due to privacy restriction.

## References

[1] Baskin T.W., Holcomb J.B. (2005). Bombs, mines, blast, fragmentation, and thermobaric mechanisms of injury. Ballistic Trauma: A Practical Guide.

[2] Yao W.J., Wang X.M., Li W.B. (2010). Effect of metal powder on blast power of the low collateral damage ammunition. Adv. Mater. Res..

[3] Loiseau J., Pontalier Q., Milne A.M., Goroshin S., Frost D.L. (2018). Terminal velocity of liquids and granular materials dispersed by a high explosive. Shock Waves.

[4] Pontalier Q., Loiseau J., Goroshin S., Frost D.L. (2018). Experimental investigation of blast mitigation and particle–blast interaction during the explosive dispersal of particles and liquids. Shock Waves.

[5] Kyner A., Dharmasena K., Williams K., Deshpande V., Wadley H. (2017). High intensity impulsive loading by explosively accelerated granular matter. Int. J. Impact Eng..

[6] Chen X., Li X., Yan H., Wang X., Miao Y. (2017). Explosive compact-coating of tungsten–copper alloy to a copper surface. Mater. Res. Express.

[7] Bai C.H., Chen Y.H., Li J.P., Wang Z.Q., Liu L. (2010). Charge forms for explosion dispersal of metal particles. Explos. Shock Waves.

[8] Li W., Yao W., Zhu W., Li W., Gao D., Tian S., Han C., Liu Y. (2024). Penetration of ballistic gelatin by explosion-driven inert metal particles. Lat. Am. J. Solids Struct..

[9] Guo M., Li X., Chen Y., Wang H. (2024). Microscopic chemical reaction mechanism and kinetic model of Al/PTFE. Polymers.

[10] Wu J.X., Fang X., Gao Z.R., Wang H.X., Huang J.Y., Wu S.Z., Li Y.C. (2018). Investigation on mechanical properties and reaction characteristics of Al-PTFE composites with different Al particle size. Adv. Mater. Sci. Eng..

[11] Wu J., Wang H., Fang X., Li Y., Mao Y., Yang L., Yin Q., Wu S., Yao M., Song J. (2018). Investigation on the thermal behavior, mechanical properties and reaction characteristics of Al-PTFE composites enhanced by Ni particle. Materials.

[12] Feng B., Li Y.C., Wu S.Z., Wang H.X., Tao Z.M., Fang X. (2016). A crack-induced initiation mechanism of Al-PTFE under quasi-static compression and the investigation of influencing factors. Mater. Des..

[13] Wang L., Liu J., Li S., Zhang X. (2016). Investigation on reaction energy, mechanical behavior and impact insensitivity of W–PTFE–Al composites with different W percentage. Mater. Des..

[14] Ding L., Cui X., Tang W., Zhong X., Zhao Y., Huang Y., Shi P., Xue X. (2022). Research on the constitutive model of PTFE/Al/Si reactive material. Polymers.

[15] Sun T., Liu A., Ge C., Yuan Y., Wang H. (2022). Mechanical properties, constitutive behaviors and failure criteria of Al-PTFE-W reactive materials with broad density. Materials.

[16] Tang E., Sun Z., Li L., Peng H., Han Y., Chen C., Chang M., Guo K., He L. (2023). Dynamic constitutive model and ignition behavior of enhanced Al/PTFE. Int. J. Impact Eng..

[17] Gao G., Tang E., Yang G., Han Y., Chang M., Guo K., He L. (2024). Parameter determination and verification of ZWT viscoelastic dynamic constitutive model of Al/Ep/W material considering strain rate effect. Int. J. Impact Eng..

[18] Kumar D., Ruan D., Khaderi S.N. (2023). Triaxial characterization of foams at high strain rate using split-Hopkinson pressure bar. Exp. Mech..

[19] Bandaru A.K., Chouhan H., Prasad S., Weaver P.M., Bhatnagar N., O’Higgins R. (2022). Effect of weave pattern on high strain rate performance of glass/polytetrafluoroethylene composites. Polym. Compos..

[20] Tang E., Li S., Chen C., Han Y. (2020). Dynamic compressive behavior of fiber reinforced Al/PTFE active materials. J. Mater. Res. Technol..

[21] Frew D.J., Forrestal M.J., Chen W. (2005). Pulse shaping techniques for testing elastic-plastic materials with a split Hopkinson pressure bar. Exp. Mech..

[22] Chen X., Ge L., Zhou J., Wu S. (2016). Experimental study on split Hopkinson pressure bar pulse-shaping techniques for concrete. J. Mater. Civ. Eng..

[23] Vecchio K.S., Jiang F. (2007). Improved pulse shaping to achieve constant strain rate and stress equilibrium in Split-Hopkinson pressure bar testing. Metall. Mater. Trans. A.

[24] Ameri A.A.H., Brown A.D., Ashraf M., Hazell P.J., Quadir M.Z., Escobedo-Diaz J.P. (2019). An effective pulse-shaping technique for testing stainless steel alloys in a Split-Hopkinson pressure bar. J. Dyn. Behav. Mater..

[25] Meng H., Li Q.M. (2003). Correlation between the accuracy of a SHPB test and the stress uniformity based on numerical experiments. Int. J. Impact Eng..

[26] Korthäuer M., Ataya S., El-Magd E. (2006). Effects of deformed volume, volume fraction and particle size on the deformation behaviour of W/Cu composites. Theor. Appl. Fract. Mech..

[27] Luo J.R. (2001). Study on Damage, Fracture and Constitutive Relation of PBX.

[28] Reaugh J. (2011). HERMES: A Model to Describe Deformation, Burning, Explosion, and Detonation.

[29] Bennett J.G., Haberman K.S., Johnson J.N., Asay B.W. (1998). A constitutive model for the non-shock ignition and mechanical response of high explosives. J. Mech. Phys. Solids.

[30] Tang Z.P., Tian L.Q., Zhu Z.X. Research on mechanical properties of epoxy resin at high strain rates. Proceedings of the 2nd National Conference on Blast and Impact Mechanics.

